# Supplementary material for: Learning curve and surgical outcome of robotic assisted colorectal surgery with ERAS program
Source: Sci Rep. 2022 Nov 29;12:20566. doi: 10.1038/s41598-022-24665-w (PMC9709162; doi:10.1038/s41598-022-24665-w)
Supplement: Supplementary file 2 — Supplementary Information 2. [file 41598_2022_24665_MOESM2_ESM.zip › RAL Raw data-Ñ[▒K/106/HS_ñw╝╨░O▒Kñσ.pdf]

正式摘要(電子簽章：000000223941591)

W52 / W52 ☐ 英文標題(注意:無英文姓名-請補病人基本檔!) ☐【讀取】憑證

臺中榮民總醫院

出院病歷摘要

轉入號： 轉出號：

[redacted]

(8)流水編號	
(9)入院日期	110/08/16 (2021) 胃腸肝膽科 W52 - 111
(10)轉科日期	110/08/17 (2021) - 直腸外科
(11)出院日期	110/08/30 (2021) 住院天數計 14 日

診斷

- 入院 :A-colon cancer with liver metastasis.
- 出院 :A-colon cancer with liver metastasis

主訴

- poor apptite, epigatric pain, abdomen cramping on and off for about 3 weeks

病史

- The 61 y/o male with hx of resolved HBV. He is a 漢翔總務事人員. ADL total independent, no smoking history, drink whisky 50ml per day for decades, quitted for 3 months. Family history: father rectum cancer.
  He accepted moderna vaccine on 2021/07/07 with fever for 3 days, dry cough and muscle soreness. No fever or chills, no travel hx for recently 2 months. He came to LMD and symtpms control medicine administered. However, poor appetite and BWL from 80 to 73 kg within 2 weeks, epigatric pain intermittent and abdomen cramping progression. No obvious exacerbating factors or Relieving factors. No nause, no vomitting , no tarry, no bloody stool, but watery diarrhea 1-2 times per day.
  Due to the above reason, he came to GI LMD where abdomen sonography showed liver tumors r/o metastasis abd refered to our GI OPD. The tumor markers and abdomen CT done, which showed CA-199 12957 Unit/ml, CEA 5215 ng/ml, abdomen CT showed liver tumors with A-colon wall thick r/o colorectal cancer. Therefore, he admitted for further evaluation and treatment.
  He admitted with fair spirit, no obvious abdomen tenderness, no pain tenderness, no palpable LN, fever without obvious discommfort.
  He came to 東山休息站, 嘉義 on 08/13.

體檢發現

-
  - General appearance: A well-nourished person, with acute ill looking. Consciousness: Alert Pupil size: 3/3 mm Light reflex:
    - Normal light reflex

    Vital signs:

date	體溫(BT)	脈搏(P)	呼吸(R)	血壓(BP)	身高(BH)	體重(BW)	BMI
20210816	38℃	100次/分	19次/分	118/74mmHg	177cm	73.7kg	23.5
  - Skin
    - Normal skin turgor , No jaundice , No cyanosis , No rash , No wound / ulcer / scar
  - Lymphadenopathy
    - No lymphadenopathy
  - HEENT
    - Head: No shape deformity , No hair change , Normal lip
    - Eyes: ＊Conjunctiva: Normal , ＊Sclera: Not icteric , ＊Cornea: Clear , ＊Eyelids: Normal
    - Ears: No deformity
    - Nose: Normal appearance
    - Mouth and throat: No oral mucosa change , No throat injection , No tonsil enlargement
  - Neck
    - No thyroid enlargement , Neck: Jugular vein
  - Chest
    - Inspection: Normal and symmetric respiratory movement , No deformity , Normal breast
    - Palpation: No palpable mass , No tender point
    - Auscultation: No rales , No rhonchi , No wheezing , Chest / Auscultation / Stridor No stridor , Not perform , No friction rub
  - Heart
    - Inspection: No visible apical impulse
    - Palpation: Not palpable PMI , No thrill , No heave
    - Auscultation: Regular heart beats , No S3 , No S4 , No murmur
  - Abdomen
    - Inspection: No distension , No striae
    - Auscultation: Normal bowel sound ,
    - Palpation: No hepatomegaly , No tenderness , No rebounding pain , No palpable mass , No splenomegaly
    - Percussion: No shifting dullness
  - Genito-anus patient refuse
  - Back and spine
    - No deformity , No knocking pain
  - Extremities
    - No deformity , No clubbing finger , No pitting edema , Normal joint , Normal peripheral pulse
  - Neurological examination
    - No mental status change
    - Normal cranial nerve
    - Normal meninges
    - Normal motor
    - Normal sensory
    - Normal cerebellum
    - Normal Stance & Gait

手術日期及方法

- 手術日期及方法20210820 Robot Assisted Colectomy(R)

住院治療經過

- This is a 61 year-old man was admitted due to A-colon cancer with liver metastasis. After admission, robotic asssisted colectomy was performed on 08/19. After the surgery patient condition was smooth with diet, however, several day after the surgery, abdominal fullness was noted. KUB showed ileus. Due to those, NG decompression, NPO with adequated hydration was given. NG was removed gradually, and re-on diet step by step. Penrose drain was removed was removed today. Abdominal fullness condition slightly improved. Under stable condition, he was discharge today with medication. Advised OPD follow up.

合併症

- Nil

一般檢查

- 累積報告 :

DATE	TIME	ALB	TP	BIL.T	BIL.D	ALKP	AST	ALT	LDH	AMY	LIPASE	NH3	Ferritin	UA
1100816	1639			0.43				28	813	42	21			
1100817	0325												607.62	
1100819	0854			0.28	0.13		91	35						

•

1100820	0344	2.5		0.27	0.12		95	44						
1100823	0854	2.4		0.54				32						
1100826	0845	2.6		1.40	0.76		57	25						
1100830	0925	3.3		2.07	0.98									

DATE	TIME	WBC	RBC	HGB	HCT	MCV	PLT	BLST	NEUT	BAND	SEG	LYM	MON	EOS	BAS
1100816	1637	5610		10.1			305		68.2			18.5	10.9	2.0	0.4
1100817	0324			9.0											
1100819	0906	4620		10.2	29.9		255		65.6			24.0	10.2	0.0	0.2
1100820	0344	4900		9.9	30.5	88.2	180				88.0	10.0	2.0	0.0	0.0
1100823	0922	6590		10.7	33.0		274		75.1			17.6	5.9	1.1	0.3
1100826	0908	9500		9.7						1.0	72.0	13.0	10.0	2.0	0.0

DATE	TIME	NA	K	CL	BUN	CREAT	eGFR	CA	Mg	P	CRP	ESR	PCT	D-Dimer	LACTATE
1100816	1639	132	3.9	98	11	0.89	92.36	8.4							
1100817	0324												0.169		
1100819	0803														17.5
1100819	0854	131	4.1		6	0.88	93.57								
1100819	1046								1.9	2.2					
1100820	0344	135	4.1		7	0.69	123.9	7.8							21.3
1100823	0733														26.1
1100823	0854	129	3.8		14	0.73	116.1	7.7			17.173				
1100826	0845	133	4.2		9	0.59		7.9			15.267				
1100826	0854														17.4
1100830	0703														9.6
1100830	0925	130	5.2												

DATE	TIME	GLUCOSE	HbA1C	CHOL	TG	HDL	LDL	KETONE	CK	CKMB	TRO-T	PT-P	APTT-P	NT-pro BNP
1100816	1637											12.1	31.2	
1100816	1639								209					
1100816	1643		5.7											
1100817	1617	94												
1100818	1503											11.4	33.2	
1100819	1046			155	102									

DATE	TIME	SP-GR	PH	PROT	GLUC	KETON	BILI	UROB	OB	NITR	LEU	RBC	WBC	EPCEL
1100816	2207	1.007	6.5	Negative	Negative	Negative	Negative	4.0	Negative	Negative	Negative	0-2	0-2	0-1

DATE	TIME	PH	BEB	SCVO2	PO2	HCO3	HGB	PCO2	TCO2
1100816	1637	7.374	-2.8	64.1	34.6	22.0		38.6	23.2

• 放射線報告：檢驗項目 **:ABDOMEN (KUB),SUPINE** 報告日期 **:1100824**

主 訴：
Result:N
Finding:
> Some gaseous bowel loop dilatation in the abdomen, R/I ileus
> Bilateral psoas shadows and kidney contours are partially obscured by bowel gas
> Degenerative change of L-spine with osteophyte formations
> On nasogastric tube
> Surgical autosuture retention and drainage tube over right abdomen
Impression:
As above description.

檢驗項目 **:ABDOMEN (KUB),SUPINE** 報告日期 **:1100823**

主 訴：
Result:N
Finding:
> Marked gaseous bowel loop dilatation in the abdomen, R/I bowel obstruction or ileus
> Bilateral psoas shadows and kidney contours are partially obscured by bowel gas
> Degenerative change of L-spine with osteophyte formations
> Surgical autosuture retention and drainage tube over right abdomen
Impression:
As above description.

檢驗項目 **:CHEST AP VIEW (PORTABLE)** 報告日期 **:1100827**

主 訴：
Result:N
Finding:
> The cardiac size is within normal limit
> Mild prominent bilateral hila
> Slightly increased infiltration over bilateral lungs
> On central venous catheter, On EKG leads
> Degenerative change of thoracic and lumbar spine with osteophytes formation
Impression:
As above description.

檢驗項目 **:CHEST PA VIEW** 報告日期 **:1100820**

主 訴：
Result:N
Finding:
> No cardiomegaly
> Bilateral hilar prominence.
> Mild increased interstitial infiltration in bilateral lungs.
> Degenerative change of thoracic and lumbar spine with osteophytes formation.
Impression:
As above description.

檢驗項目 **:CHEST PA VIEW** 報告日期 **:1100825**

主 訴：
Result:N
Finding:
> No cardiomegaly
> Bilateral hilar prominence.
> Mild increased interstitial infiltration in bilateral lungs.
> Degenerative change of thoracic and lumbar spine with osteophytes formation.
Impression:
As above description.

檢驗項目 **:CT ABDOMEN-SURVEY** 報告日期 **:1100816**

主 訴：
Result:Y
Finding:
Imaging Report Form for Colorectal Carcinoma (AJCC 8th)
1. Imaging Date: 20210812
Imaging Modality, Imaging by
CT scan
Imaging Protocol: CT scan of abdomen with IV and oral contrast enhancement:
2. Tumor location / Size
Location:
Ascending
Size:
Measurable: 6.2 cm (largest diameter)
3. Tumor invasion
Tumor invades visceral peritoneum or adjacent organs
4. Regional nodal metastasis
No

- 5. Distant metastasis (In this study)
  Single organ
  6. Other findings: 1. Asymmetrical wall thickening about 6.2cm in size at A-colon with stranding of pericolic fat plane, several small pericolic lymph nodes, involving ileocecal valve, causing intestinal obstruction, R/I colon cancer.
  2. lobulated poor enhancing nodules & masses upto 13.1cm in size over both lobes, R/I liver metastases.
  3. Patent main portal vein and its major branches.
  4. slight wall thickening of gallbladder wall with several gallbladder stones and no distension of gallbladder, chronic cholecystitis could not be ruled out.
  5. Small bilateral renal stones.
  6. Presence of accessory spleen.
  7. pancreas, spleen are without specific abnormality.
  8. Small lymph nodes less than 1cm in size over para-aortic region are noted.
  Impression:
  1. R/I A-colon cancer, involving ileocecal valve, with liver metastases.
  2. Other findings, as above description

特殊檢查

- 檢驗項目 **:CT ABDOMEN-SURVEY** 報告日期 **:1100816**
  主 訴：
  Result:Y
  Finding:
  Imaging Report Form for Colorectal Carcinoma (AJCC 8th)
  1. Imaging Date: 20210812
  Imaging Modality, Imaging by
  CT scan
  Imaging Protocol: CT scan of abdomen with IV and oral contrast enhancement:
  2. Tumor location / Size
  Location:
  Ascending
  Size:
  Measurable: 6.2 cm (largest diameter)
  3. Tumor invasion
  Tumor invades visceral peritoneum or adjacent organs
  4. Regional nodal metastasis
  No
  5. Distant metastasis (In this study)
  Single organ
  6. Other findings: 1. Asymmetrical wall thickening about 6.2cm in size at A-colon with stranding of pericolic fat plane, several small pericolic lymph nodes, involving ileocecal valve, causing intestinal obstruction, R/I colon cancer.
  2. lobulated poor enhancing nodules & masses upto 13.1cm in size over both lobes, R/I liver metastases.
  3. Patent main portal vein and its major branches.
  4. slight wall thickening of gallbladder wall with several gallbladder stones and no distension of gallbladder, chronic cholecystitis could not be ruled out.
  5. Small bilateral renal stones.
  6. Presence of accessory spleen.
  7. pancreas, spleen are without specific abnormality.
  8. Small lymph nodes less than 1cm in size over para-aortic region are noted.
  Impression:
  1. R/I A-colon cancer, involving ileocecal valve, with liver metastases.
  2. Other findings, as above description

病理報告

- 活體組織切片病理報告　手術日期：**110/08/20**　病理號：**S1025775**

  診斷醫師:土仟卿 報告日期:110/08/25
  Pathologic diagnosis:
  1. Colon, terminal ileum, and appendix, robotic-assisted colectomy
  --- Adenocarcinoma, moderately differentiated, of ascending colon.
  Tumor invaded to peritoneum and metastasized to mesocolic lymph nodes (5/9). The appendix, ileocecal valve, bilateral cut ends are free of tumor.
  2. Appendix, robotic-assisted colectomy --- Unremarkable.
  Ancillary study for diagnosis:
  IHC stains for PMS2 and MSH6 are done on section F. Both above markers are preserved.
  Prognostic and predictive factor:
  1. Tumor size: 7 cm.
  2. Depth of invasion: Visceral peritoneum (including tumor continuous with serosal surface through area of inflammation).
  3. Histologic grade: G2: Moderately differentiated.
  4. Angiolymphatic invasion: Present.
  5. Perineural invasion: Not identified.
  6. Tumor deposits (discontinuous extramural extension): Present, specify number of deposits: 5.
  7. Macroscopic tumor perforation: Not identified.
  8. Surgical Margins: a) Proximal Margin: Uninvolved by invasive carcinoma, distance of tumor from margin: 14 cm. b) Distal Margin: Uninvolved by invasive carcinoma, distance of tumor from margin: 9 cm. c) Radial or Mesenteric Margin: Uninvolved by invasive carcinoma, distance of tumor from margin: 1.5 cm.
  9. Lymph node status: Involved (5/9).
  10. Extranodal involvement: Present.
  11. Tumor regression grading S/P CCRT: No known presurgical therapy.
  12. Type of polyp in which invasive carcinoma arose: Not identified.
  13. Additional pathologic findings: Not identified.
  14. Pathological TNM Stage: pT4aN2a(According to the eighth edition, American Joint Committee on Cancer Staging Guidelines for Tumors).
  15. TNM descriptors: Not applicable.
  Gross description:
  The specimen consists of 1) a segment of colon, 13.5 cm in length, and 7 cm in proximal circumference, 3 cm in distal cut end circumference, and a segment of terminal ileum, 14.5 cm in length, and 2 cm in cut end circumference. An unremarkable appendix, 5.5x0.5 cm, is noted. A gray tan firm annular elevated and centrally ulcerative tumor, 7x5.2 cm, is noted over the cecum and near the ileocecal valve, 9 cm to the nearest cut end. Attached omentum, 29x10x1 cm, is included, with several white firm nodular lesions, up to 1.8x1.3
  cm. On cross section, the tumor invaded to the mesocolon with 15 mm to the radial margin grossly, and serosal retraction is also noted.
  The appendix, ileocecal valve, and both cut ends are free of tumor.
  2) "appendix", a piece, 2.4x0.8x0.4 cm. Representative part for section: A) proximal cut end B) distal cut end C) appendix D) ileocecal valve E-G) tumor H) colon L-N) lymph nodes; all for section: K) specimen 2.
  Note:
  1. The interpretation of MMR panel and recommended studies are as below: (1) No loss of nuclear expression of MMR proteins: low probability of microsatellite instability-high (MSI-H) . (2) Loss of

- nuclear expression of MLH1 and PMS2: testing for methylation of the MLH1 promoter and/or mutation of BRAF is indicated (the presence of a BRAF V600E mutation and/or MLH1 methylation suggests that the tumor is sporadic and germline evaluation is probably not indicated; absence of both MLH1 methylation and of BRAF V600E mutation suggests the possibility of Lynch syndrome, and sequencing and/or large deletion/duplication testing of germline MLH1 may be indicated). (3) Loss of nuclear expression of MSH2 and MSH6: high probability of Lynch syndrome (sequencing and/or large deletion/duplication testing of germline MSH2 may be indicated, and, if negative, sequencing and/or large deletion/duplication testing of germline MSH6 may be indicated). (4) Loss of nuclear expression of MSH6 only: high probability of Lynch syndrome (sequencing and/or large deletion/duplication testing of germline MSH6 may be indicated). (5) Loss of nuclear expression of PMS2 only: high probability of Lynch syndrome (sequencing and/or large deletion/duplication testing of germline PMS2 may be indicated). (6) Please note: There are exceptions to the above IHC interpretations. These results should not be considered in isolation, and clinical correlation with genetic counseling is recommended to assess the need for germline testing.
  2. This case has been peer reviewed by two doctors.
  Erratum: 2021/08/27.
  Prognostic and predictive factor:
  14. Pathological TNM Stage: pT4aN2aM1c(According to the eighth edition, American Joint Committee on Cancer Staging Guidelines for Tumors).
  Gross description:
  Representative part for section: A) proximal cut end B) distal cut end C) appendix D) ileocecal valve E-G) tumor H) colon J) omentum mass L-N) lymph nodes; all for section: K) specimen 2.
  Comment:
  Section J represented omentum mass and not located within the territorial lymphatic drainage of mesocolon. Therefore, metastasis (M1 disaese) is considered.
  #T-59420_2 #M-81403_2 2 1105 S0000F
  #T-C4560_2 #M-81403_2 2 1105 000000
  #T-59200_2 #M-09183_2 0 1105 000000
  #T-D4600_2 #M-81406_2 0 1105 ER0000

其它

- 檢驗項目 :**Sonography** 報告日期 :**1100819**
  Result:
  Findings:
  Liver: mild coarsening of liver parenchyma with mild increase brightness, multiple hyperechoic lesion in the gallbladder, some parenchyma was blocked by bowel gas
  GB: Multiple 7.1 mm hyperechoic lesion with acoustic shadow inside gallbladder
  CBD: no evidence dilatation
  IHD/CHD: no evidence of dilatation
  Pancreas: Partial blocked by bowel gas
  Kidney: bilateral normal size without focal lesion
  Spleen: not enlarged
  Sonography diagnosis
  1. Multiple hyperechoic lesion over bilateral lobes of liver, 112.1*68.9 mm suspect colon cancer liver metastasis
  2. Gallbladder stones, 7.1 mm
  Suggestion: follow up sonography regularly

  檢驗項目 :**R15 HEPATIC BLOOD FLOW ESTIMATE** 報告日期 :**1100817**
  Result:
  RESULT

Fraction retained plasma dye disappering per minute	16.90	%
T 1/2	4.1	min
15'th minute retention rate	7.80	%
Estimate hepatic blood flow	----	ml/min

出院時情況

- 3.改門診治療

出院指示

藥名	劑量	劑量單位	用法	頻次	數量	數量單位	現況	天數	單位
Medicon-A cap 20mg	1	CAP	PO	BID&HS	15	CAP	TH	5	天
Through tab	1	TAB	PO	QN	8	TAB	TH	8	天
C.B. oint 5gm		TUB	TOP	TID	0	TUB	TH	1	瓶
Tamso SR cap 0.2mg	1	CAP	PO	QD	8	CAP	TH	8	天
Ultracet tab	1	TAB	PO	Q6H	20	TAB	TH	5	天
Stilnox tab 10mg	1	TAB	PO	HS	8	TAB	TH	8	天
Mag. Oxide tab	2	TAB	PO	TID	48	TAB	TH	8	天
Spironolactone-25	1	TAB	PO	QD	5	TAB	TH	5	天
Econ tab 40mg	1	TAB	PO	QID	20	TAB	TH	5	天

出院計劃

主治醫師 2062F 蔣鋒帆 / 住院醫師 2365H 蘇晏中

正式摘要(電子簽章：000000223941591)

W52 / W52

doctorRank:B
